# Supplementary material for: Chromothripsis during telomere crisis is independent of NHEJ, and consistent with a replicative origin
Source: Genome Res. 2019 May;29(5):737–49. doi: 10.1101/gr.240705.118 (PMC6499312; doi:10.1101/gr.240705.118)
Supplement: Supplemental Material [file supp_gr.240705.118_Supplemental_file_1.zip › contigs/annotated_contigs/DB57/contig.2.DB57_length_354_mean_cov_20.4124293785.docx]

**DB57_length_354_mean_cov_20.4124293785**

```
CAGTCCACCCTCTGCCTGAGCGGGC|GGTCTCGGGTGCGACTGAGCTTCTGACCCTTTGAGAGGAGTCAGGGACACGCAGCGTCTCCAG
                          >chr8:144119923-144120161 - E=5e-106 p=0e+00
CAAGCTGGTGGCAGAAACTGATGGAGCATCCTAATCACAAAATAGGAAGAGAGTTTGCAAAATAGGAGCATTTACCAGAAGCCTCTCTC

TCTCCCTCCCTCCCTCTCTCCCTCTCTCTCTCTGTCTCCCTCCCTCCCTCCCTCTCTCTCTCTCTCTCCCTCTCTCTCTCTCTCTCTC|

CCTCCCCCCCC|CTCCCCCCCCCCCCCCCGTCTCTCTCTCTCTC|CCCTCTCCTCCCCCCCGACCTTTTCTTTCCCGTTCAAGCATACC
           >chrX:129694542-129694574 + E=9e-02
TG
```
